# Role of lung ornithine aminotransferase in idiopathic pulmonary fibrosis: regulation of mitochondrial ROS generation and TGF-β1 activity

**DOI:** 10.1038/s12276-024-01170-w

**Published:** 2024-02-28

**Authors:** Jong-Uk Lee, Ki Sung Song, Jisu Hong, Hyesun Shin, Eunji Park, Junyeong Baek, Shinhee Park, Ae-Rin Baek, Junehyuk Lee, An Soo Jang, Do Jin Kim, Su Sie Chin, U-Jin Kim, Sung Hwan Jeong, Sung-Woo Park

**Affiliations:** 1 Division of Allergy and Respiratory Medicine, Department of Internal Medicine, Soonchunhyang University Bucheon Hospital, Bucheon, 14584 Gyeonggi-Do South Korea; 2 Department of Pathology, Soonchunhyang University Bucheon Hospital, Bucheon, 14584 Gyeonggi-Do South Korea; 3 Department of Internal Medicine, Environmental Health Center Kangwon National University, Gangwondaehakgil, Chuncheon-si, Gangwon-do South Korea; 4 Department of Allergy, Pulmonary and Critical Care Medicine, Gachon University, Gil Medical Center, Incheon, South Korea

**Keywords:** Prognostic markers, Molecular biology

## Abstract

Idiopathic pulmonary fibrosis (IPF) is characterized by aberrant lung remodeling and the excessive accumulation of extracellular matrix (ECM) proteins. In a previous study, we found that the levels of ornithine aminotransferase (OAT), a principal enzyme in the proline metabolism pathway, were increased in the lungs of patients with IPF. However, the precise role played by OAT in the pathogenesis of IPF is not yet clear. The mechanism by which OAT affects fibrogenesis was assessed in vitro using OAT-overexpressing and OAT-knockdown lung fibroblasts. The therapeutic effects of OAT inhibition were assessed in the lungs of bleomycin-treated mice. OAT expression was increased in fibrotic areas, principally in interstitial fibroblasts, of lungs affected by IPF. OAT levels in the bronchoalveolar lavage fluid of IPF patients were inversely correlated with lung function. The survival rate was significantly lower in the group with an OAT level >75.659 ng/mL than in the group with an OAT level ≤75.659 ng/mL (HR, 29.53; *p* = 0.0008). OAT overexpression and knockdown increased and decreased ECM component production by lung fibroblasts, respectively. OAT knockdown also inhibited transforming growth factor-β1 (TGF)-β1 activity and TGF-β1 pathway signaling. OAT overexpression increased the generation of mitochondrial reactive oxygen species (ROS) by activating proline dehydrogenase. The OAT inhibitor L-canaline significantly attenuated bleomycin-induced lung injury and fibrosis. In conclusion, increased OAT levels in lungs affected by IPF contribute to the progression of fibrosis by promoting excessive mitochondrial ROS production, which in turn activates TGF-β1 signaling. OAT may be a useful target for treating patients with fibrotic lung diseases, including IPF.

## Targeting OAT: a potential therapeutic pathway for fibrotic lung disease

Idiopathic pulmonary fibrosis (IPF, a chronic lung disease marked by excessive buildup of extracellular matrix or ECM, substances that provide structural support to cells, in the lungs) is still not fully understood, especially the role of ornithine aminotransferase (OAT, an enzyme that helps convert ornithine into proline). This study found that OAT was more present in the lungs of IPF patients, especially in areas with severe fibrotic (excessive tissue) remodeling. Increased OAT led to more fibroblast (cells that produce collagen and other fibers) growth, while less OAT reduced it. The study concluded that high OAT levels in IPF lungs contribute to fibrosis progression by enhancing TGF-β1 (a protein that controls cell growth and division) activity and increasing mitochondrial ROS generation. The findings suggest that stopping OAT may reduce fibrosis, offering a potential new treatment for IPF. This summary was initially drafted using artificial intelligence, then revised and fact-checked by the author.

## Introduction

Idiopathic pulmonary fibrosis (IPF) is a chronic progressive lung disease that is characterized by alveolar epithelial cell (AEC) hyperplasia, increased myofibroblast numbers, interstitial extracellular matrix (ECM) deposition, and lung architecture remodeling^1^. The excessive deposition of heterogeneously distributed ECM components in the alveolar parenchyma is a key characteristic of IPF. The ECM consists of an interlocking network of fibrous proteins and glycosaminoglycans, but the most abundant ECM component in most tissues is collagen.

Myofibroblasts, which are modified contractile fibroblasts, alter other ECM proteins and regulate collagen metabolism^2^. The production, deposition, and remodeling of collagen in the ECM is a dynamic process^3^. In normal lungs, the degradation and synthesis of collagen and other ECM proteins are carefully balanced^3^, and disruption of collagen homeostasis can contribute to fibrotic lung disease^4,5^. The glycoprotein fibronectin mediates cell-matrix adhesion by binding ECM molecules, such as collagen, and cell-surface integrins^6^. Fibronectin is particularly abundant in the lungs of patients with IPF and is essential for myofibroblast differentiation^6^. Alpha-smooth muscle actin (α-SMA) is often used as a marker of contractile myofibroblasts, and α-SMA^+^ cells are considered to be activated fibroblasts that are capable of overproducing collagen^7,8^.

Transforming growth factor (TGF)-β1 plays an important role in myofibroblast activation and differentiation. In patients with IPF, TGF-β1 expression is particularly high in fibrotic areas of the lungs, and TGF-β1 drives the expression of genes that encode key ECM components, including collagen, fibronectin, and α-SMA^9–11^. Activated TGF-β1 ligands are potent drivers of ECM deposition and show natural affinities for the ECM; thus, these ligands contribute to the generation of a profibrotic environment at the site of injury^9^. Therefore, regulation of TGF-β1 activity may be a useful therapeutic strategy for treating patients with diseases that involve ECM protein overproduction, such as IPF^12^.

Ornithine aminotransferase (OAT) is a key enzyme that is involved in converting ornithine into proline^13^. OAT can convert L-ornithine into pyrroline-5-carboxlate, which is further metabolized into L-proline to synthesize collagen^14^. Several years ago, we reported that the levels of proline, the proline-to-ornithine ratio, and the mRNA expression of OAT were increased in patients with IPF, as shown by analyses of publicly available lung-tissue microarray datasets from another cohort that included 50 control subjects and 119 patients with IPF^15,16^. However, the role of OAT in IPF remains unclear.

In the present study, we investigated the role of OAT in fibrogenesis in patients with IPF. Lung tissue from patients with IPF was used to study the expression of OAT. Cell culture experiments were performed to determine the mechanism by which OAT expression was regulated, and the therapeutic effects of inhibiting OAT were evaluated using bleomycin (BLM)-induced fibrotic lung models.

## Materials and methods

### Human samples

All the human lung tissues and bronchoalveolar lavage (BAL) fluid samples from patients with IPF and control subjects were obtained from the Biobank at Soonchunhyang University Bucheon Hospital (Bucheon-si, South Korea). IPF diagnoses were based on an international consensus statement by the American Thoracic Society and the European Respiratory Society. Histological diagnoses of usual interstitial pneumonia were confirmed with surgical lung biopsy specimens. The control samples were normal lung specimens that were not affected by disease, and these samples were obtained from patients who underwent surgery to treat lung cancer. All the patients were recruited between January 2011 and January 2016. This protocol was approved by the local ethics committee of Soonchunhyang University Bucheon Hospital (SCHBC_IRB_2016–12–024–002).

### Immunohistochemical staining

Lung tissues were dehydrated and embedded in paraffin. For histological examination, 4-µm-thick tissue sections and BALF cells on slides were treated with 1.4% H_2_O_2_–methanol for 30 min to block endogenous peroxidase. Then, nonspecific binding was blocked with 1.5% normal serum, and the slides were incubated with rabbit anti-OAT polyclonal antibodies (1:200; Abcam, Cambridge, UK, #ab137679). The next day, the sections were incubated with ABC kit reagents (Vector Laboratories, Burlingame, CA, USA). The color reaction was developed by incubation with a liquid 3,3′-diaminobenzidine positive-substrate kit (Golden Bridge International, Inc., Mukilteo, WA, USA). After immunohistochemical staining, the slides were counterstained with Harris’s hematoxylin for 1 min.

### Fibroblast isolation and culture

Primary human lung fibroblasts were obtained from Soonchunhyang Biobank (Bucheon, South Korea) as previously described^17^. Briefly, fibroblasts were isolated from lung tissues that were harvested from IPF patients via video-assisted thoracoscopic biopsy. The lung samples were obtained by biopsy usually 1 week after hospital admission. No patient had been treated with corticosteroids or immunosuppressive drugs at the time of biopsy. Control fibroblasts were obtained from individuals who underwent lobectomies for the removal of primary lung tumors. No morphological evidence of disease was observed in the tissue samples that were used to isolate the control cells. Lung fibroblasts were isolated from IPF or control specimens via mechanical dispersal and trypsin digestion and minced into 1-mm^2^ fragments. Fibroblast cultures were established in Dulbecco’s modified Eagle’s medium (DMEM) supplemented with 10% (v/v) fetal bovine serum (FBS, Thermo Fisher Scientific, Rockford, IL, USA), 100 U/mL penicillin, 100 mg/mL streptomycin, and 0.25 μg/mL amphotericin B (Gibco, Carlsbad, CA, USA). All the cells were cultured at 37 °C in 95% air:5% CO_2_ (v/v) until just before they reached confluence, generally for 1–2 weeks. After three passages, immunoblotting analyses were performed using anti-vimentin antibody to examine the adherent cells that were harvested from the same culture vessels. All the cells exhibited the morphological characteristics of fibroblasts. All the experiments with IPF and control fibroblasts were performed on cells before passage 5. The fibroblasts were stimulated for 24 h in serum-free medium supplemented with 0.1% (v/v) FBS, 10 µg/mL urban particulate matter (PM10; Sigma‒Aldrich, St. Louis, MO, USA), or 10 µg/mL BLM (Nippon Kayaku Co., Ltd., Tokyo, Japan). Cell lysates and culture supernatants were collected for further analysis.

### Cell culture and treatments

Human MRC5 lung fibroblasts (ATCC, Manassas, VA, USA) were cultured in DMEM supplemented with 10% (v/v) FBS (Thermo Fisher Scientific), 100 U/mL penicillin, and 100 µg/mL streptomycin (Gibco). The cells were maintained in a humidified atmosphere with 5% (v/v) CO_2_ at 37 °C. The cells were stimulated for 48 h in serum-free medium supplemented with 0.1% bovine serum albumin (BSA) and 5 ng/mL TGF-β1 or 10 µg/mL BLM.

### Overexpression of OAT

The OAT-Flag plasmid (pCMV-OAT) and its control vector (pCMV-Entry) were purchased from OriGene (Rockville, MD, USA). Each vector was transfected into MRC5 cells using Lipofectamine^®^ 2000 (Invitrogen, Carlsbad, CA, USA). Stable OAT-overexpressing cell lines were selected using 1 mg/mL G418 (Sigma‒Aldrich). Overexpression of OAT-FLAG was confirmed by immunoblotting (Supplementary Fig. 1a).

### Lentiviral transduction of MRC5 fibroblasts to knockdown OAT

Transient and stable transfection procedures were performed using small hairpin (sh)RNA lentiviral transduction particles according to the manufacturer’s instructions (Sigma‒Aldrich). An shRNA targeting the OAT gene (OAT shRNA [h]) and the corresponding control shRNA (h) (Sigma‒Aldrich) were used for RNA interference. For lentiviral transduction, MRC5 fibroblasts were cultured at 37 °C in 5% CO_2_ in 100-mm culture dishes in medium supplemented with 10% FBS (Thermo Fisher Scientific), 100 units/mL penicillin, 100 mg/mL streptomycin (Gibco), and 10 µL/mL L-glutamine (Gibco). When the cells reached 70% confluence, transduction was performed according to the manufacturer’s instructions (Sigma‒Aldrich). In brief, lentivirus expressing shRNA against human OAT (OAT knockdown) or lentivirus expressing nontargeting scrambled shRNA (scramble) was added to the cells at a multiplicity of infection (MOI) of 10. The medium was changed after 24 h. Three days after transduction, stable clones of MRC5 cells expressing OAT shRNA or scramble shRNA were selected over an additional 10 days of culture using 5 µg/mL puromycin (Sigma‒Aldrich). The medium was changed every 48 h. Gene silencing was confirmed by immunoblotting (Supplementary Fig. 1b).

### Immunoblotting

Proteins were extracted from lung tissues or cells in lysis buffer (Thermo Fisher Scientific) using proteinase and phosphatase inhibitor cocktails (Roche Diagnostics, Basel, Switzerland), followed by centrifugation. For each experiment, equal quantities of total proteins were resolved using 10% sodium dodecyl sulfate-polyacrylamide gel electrophoresis. The proteins were transferred to polyvinylidene difluoride membranes (Millipore, Billerica, MA, USA).

The membranes were subsequently blocked in 5% skim milk and incubated for 24 h at 4 °C with the following primary antibodies: anti-OAT (1:1,000; Santa Cruz Biotechnology, Inc., Santa Cruz, #SC-374243), anti-PRODH (1:1,000; Santa Cruz Biotechnology, Inc, #SC-376401), anti-collagen I (1:1000; Abcam, #ab34710), anti-fibronectin (1:1000; Abcam, #ab268020), anti-α-SMA (1:500; Abcam, #ab7817), anti-β-actin (1:10,000; Sigma‒Aldrich, #A1978), anti-Smad3, anti-pSmad3 (both 1:1000; Cell Signaling Technology, Danvers, MA, USA, #9523, #9520), anti-Smad2, anti-pSmad2 (both 1:1000; Cell Signaling Technology, #53398, #3108), anti-extracellular signal-regulated kinase (ERK)1/2, anti-pERK1/2 (both 1:1,000; Cell Signaling Technology, #9102, #9101), anti-p38 mitogen-activated protein kinase (MAPK), anti-pp38 MAPK (both 1:1000; Cell Signaling Technology, #9212S, #9211S), anti-JNK, and anti-pJNK ((both 1:1000; Cell Signaling Technology, #9252S, #9251S). After washing several times with phosphate-buffered saline containing Tween, the membranes were incubated with a horseradish peroxidase-conjugated anti-rabbit or anti-mouse immunoglobulin G (IgG) secondary antibody (GenDEPOT, Katy, TX, USA). The membranes were analyzed by chemiluminescence (Thermo Fisher Scientific and Bio-Rad, Hercules, CA, USA) using the ChemiDoc™ Touch Imaging System (Bio-Rad).

### Enzyme-linked immunosorbent assay (ELISA) analysis of OAT and proline levels

The protein concentrations of OAT in BALF samples, primary fibroblast culture supernatants, and mouse lung lysates were measured using an ELISA kit (BALF samples; Cloud Clone Corp., Houston, TX, USA, mouse lungs; Abbexa., Ltd., Cambridge, UK). The proline levels in cell lysates were also measured using an ELISA kit (MyBioSource, San Diego, CA, USA). All the assays were performed following the manufacturers’ instructions. The lower limits of detection were 0.625 ng/mL for OAT and 0.1 ng/mL for proline. Values below these limits were recorded as 0 pg/mL. The inter- and intra-assay coefficients of variance were <15%.

### Immunofluorescence and mitochondrial labeling

Primary lung fibroblasts were cultured in a dish at 37 °C, and the mitochondria were stained with 100 nM MitoTracker Red (Invitrogen) for 20 min. Then, the cells were fixed with 4% (v/v) paraformaldehyde (PFA) for 45 min, permeabilized in ice-cold buffer (0.1% [w/v] sodium citrate and 0.1% [v/v] Triton X-100 in distilled water) for 3 min, blocked with Dulbecco’s phosphate-buffered saline with 1% (w/v) BSA for 1 h; and incubated with an anti-OAT antibody (1:200; Santa Cruz, #SC-374243) overnight at 4 °C. After washing, the cells were incubated with a fluorescein isothiocyanate (FITC)-conjugated anti‐mouse antibody (1:1000; Abcam, #ab6785) for 1 h at room temperature and mounted in fluorescence medium containing 4ʹ,6-diamidino-2-phenylindole (DAPI; Abcam). To measure the expression of TGF-β1 in BLM-treated OAT-knockdown cells, the cells were subjected to double immunofluorescence staining for TGF-β1 (1:100; Thermo Fisher Scientific, #MA5-15065) and OAT (1:100). FITC-conjugated donkey anti-rabbit IgG (1:1000; Abcam, #ab150061) and PE-conjugated goat anti-mouse IgG (1:1000; Abcam, #ab97024) served as the secondary antibodies. All the stained images were captured using a Leica DMi8 confocal laser microscope (Leica Microsystems; Wetzlar, Germany).

### Proliferation assays

Cell proliferation was evaluated in triplicate by measuring the transformation of water-soluble tetrazolium salt (WST)-1 (Roche Diagnostics) into formazan by mitochondrial dehydrogenases. Stable OAT-knockdown cells were seeded in 96-well plates and incubated for 48 h. Next, the cells were stimulated with 5 ng/mL TGF-β1, and assays were performed by adding WST-1 directly to the wells and incubating the 96-well plates at 37 °C for 60 min. Then, the plates were read using a scanning multiwell spectrophotometer.

### Migration assays

Migration of OAT-knockdown MRC5 cells was investigated by seeding 20,000 cells in two wells of a wound-healing assay cell-culture insert (Ibidi, Munich, Germany). The cells were grown to confluence for 1 day before removing the insert. Images were recorded at baseline and 48 h, and the images were analyzed using ImageJ software^18^.

### Measurement of active TGF-β1

ELISA kits (R&D Systems) were used to measure the concentrations of active TGF-β1 in cell lysates and mouse lung tissues in accordance with the manufacturer’s instructions.

### Knockdown of proline dehydrogenase (PRODH)1 in stable OAT-overexpressing cells

PRODH1-specific siRNA was purchased from Bioneer (Daejeon, South Korea). Stable OAT-overexpressing cells were transfected with either siRNA targeting PRODH or scrambled siRNA at a concentration of 100 nM using an Amaxa 4D-Nucleofector unit (Lonza, Koln, Germany) in accordance with the manufacturer’s instructions. Immunoblotting analyses were performed to confirm the efficacy of siRNA-mediated PRODH knockdown.

### Measurement of mitochondrial reactive oxygen species (ROS)

DMEM-adapted MRC5 cells were seeded at a density of 20,000 cells/dish in a total of 2 mL phenol-free DMEM and grown for 3 days in the presence or absence of 10 µg/mL BLM. Then, the cells were treated with a final concentration of 5 µM MitoSOX Red (Invitrogen) in phenol-free DMEM for at least 10 min at 37 °C. The stained cells were excited at 510 nm during live confocal imaging, and the emitted fluorescence was detected at 580 nm (the FL2 channel) using a flow cytometer (Becton Dickinson Biosciences, San Jose, CA, USA).

### Animal model of BLM-induced lung fibrosis and treatment with L-canaline

Male C57BL/6 mice (Orient Bio, Inc., Charles River, Sungnam, South Korea) were maintained under pathogen-free conditions with a 12-h light period at 22 °C and 20–50% humidity. Food and water were provided ad libitum. All the animal procedures followed a protocol that was approved by the Institutional Animal Care and Use Committee of Soonchunhyang University Bucheon Hospital (SCHBC-animal-2019-03). The mice were administered 3 U/kg BLM (Nippon Kayaku Co., Ltd.) dissolved in 100 µL endotoxin-free water via intratracheal instillation to induce lung fibrosis^19^. Sham controls received an equal volume of endotoxin-free water alone. On Days 8, 9, 10, 11, and 12, the mice were anesthetized with a mixture of ketamine (Yuhan Corp., Seoul, South Korea) and xylazine (Bayer Corp., Shawnee Mission, KS, USA). Then, 100 nM L-canaline (Sigma‒Aldrich), which was dissolved in endotoxin-free water, was instilled intranasally. All the mice in the control groups were treated with endotoxin-free water alone. On Day 21, the mice were sacrificed by intraperitoneal injection of an overdose of ketamine and xylazine, and BAL was performed by instilling 1 mL phosphate-buffered saline with gentle retrieval (four times).

### Histological assays

A portion of each left lung was fixed in 4% (v/v) buffered paraformaldehyde and embedded in paraffin. The tissues were cut into 5-µm-thick sections and stained with H&E or Masson’s trichrome. The right lung was snap-frozen by immersion in liquid nitrogen and stored at –80 °C prior to RNA and protein extraction. Lung sections were stained with H&E for histopathological analyses or with Masson’s trichrome to evaluate collagen content and distribution. The Ashcroft score was used to assess lung fibrosis, as previously described^20^.

### Masson’s trichrome staining of lung tissues

Lung tissues were fixed in Bouin’s solution, washed in tap water for 5 min at room temperature, and then stained for 10 min with Weigert’s iron hematoxylin. After washing in tap water, the slides were stained with a mixture of 1% acid fuchsin and 1% Biebrich scarlet in distilled water for 2 min and then treated with 2.5% phosphomolybdic-phosphotungstic acid for 10 min. The sections were stained with aniline blue for 1 min, treated with 1% acetic acid for 1 min, and then dehydrated using a graded series of ethanol washes followed by five washes in absolute ethanol. Finally, the sections were immersed in xylene and then mounted in balsam.

### Measurement of hydroxyproline levels in mouse lungs

To quantify collagen contents in the lungs, we performed hydroxyproline assays (Sigma‒Aldrich) on the right lung from each mouse in accordance with the manufacturer’s instructions. Briefly, the lungs were weighed, homogenized in sterile water, and hydrolyzed in 12 N HCl at 120 °C for 3 h. The hydrolyzed samples were incubated with 4-(dimethylamino) benzaldehyde for 90 min at 60 °C, and the absorbance of oxidized hydroxyproline was measured at 560 nm. The collagen content is expressed in micrograms per milligram of lung tissue.

### Statistical analyses

Data were analyzed using SPSS software (ver. 20.0; *SPSS*, Inc., Chicago, IL, USA). The distribution of data was assessed using the Shapiro–Wilk test. Student’s *t* test or the Mann–Whitney *U* test was used to analyze continuous data. Categorical data were compared using the χ^2^ test. Correlations between OAT levels and other parameters were analyzed using Spearman’s correlation coefficient. Receiver operating characteristic (ROC) curve analyses were used to identify OAT levels in BALF that could be used as cutoff values for predicting IPF survival. Survival was estimated by reference to the BALF samples using Kaplan–Meier survival analyses and log-rank tests. Skewed data are expressed as medians with 25% and 75% quartiles; normally distributed data are expressed as the means ± standard errors of the means. *p* < 0.05 was considered to indicate significant differences.

## Results

### Clinical characteristics

BALF samples were obtained from patients with IPF (*n* = 59) and control subjects (*n* = 20). As expected, patients with IPF had significantly lower forced vital capacity (FVC) and diffusing capacity of the lung for carbon monoxide (DLCO) values than control subjects (*p* < 0.05). The IPF group consisted of 45 survivors and 14 nonsurvivors. The nonsurvivors had lower FVC values, but all the other clinical and physiological parameters were similar between the groups (Table 1).

**Table 1:** Demographic characteristics of the study subjects.

	Control Subjects	IPF Patients		
		Total	Survivors	Nonsurvivors
No.	20	59	45	14
Age (year)	55 (51–62)	64.54 (59.53–69)	62.94 (59.03–69.1)	66.15 (55.58–68.33)
Male sex	11 (55.0)	40 (67.8)	30 (66.7)	10 (71.4)
History of Smoking	4 (20.0)	12 (20.3)	10 (22.2)	2 (14.3)
BMI, kg/m^2^	24.34 (22.24–27.3)	24.06 (22.21–26.06)	23.68 (22.19–25.97)	25.07 (20.96–26.4)
Follow-up duration (year)	—	4.06 (1.82–5)	5 (1.19–5)	3.46 (2.29–4.27)
Deaths	—	14 (23.7)	0 (0.0)	14 (100)
FVC (% pred)	85.5 (81.5–89.5)	70 (64–79)^†^	72 (64.75–83.75)^#^	64.5 (57–74)
DLCO (% pred)	89 (76.75–91.5)	64 (52–72)^†^	64.5 (57–73.75)	57 (49–66)
Treatment status				
Anti-fibrotic agents	—	20 (33.9)	17 (37.8)^#^	3 (21.4)
Anti-inflammatory agents	—	35 (59.3)	26 (57.8)	9 (64.3)
None	—	13 (22.0)	9 (20.0)	4 (28.6)

Data are presented as the median (25th–75th percentile) or number (%).

*BMI* body mass index, *FVC* forced vital capacity, *DLco* diffusing capacity of the lung for carbon monoxide.

^†^*P* < 0.05 compared with control subjects, ^#^*P* < 0.05 compared with nonsurvivors.

### Increased OAT expression in the lungs of patients with IPF and BLM-treated mice

**Fig. 1:**
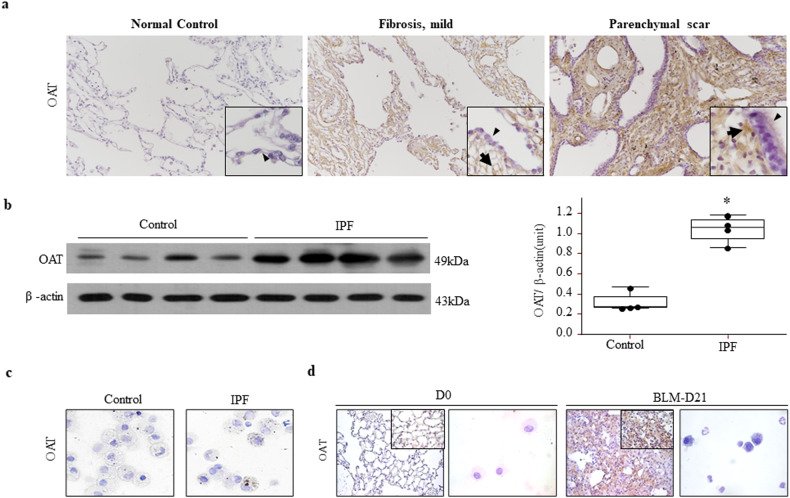
Ornithine aminotransferase (OAT) expression is increased in the lungs of patients with idiopathic pulmonary fibrosis (IPF). **a** Immunohistochemical staining for OAT in the lungs of control subjects compared to patients with IPF. Original magnification 100×; inset magnification 1000×. The arrowheads indicate alveolar epithelial cells (AECs) in the normal control tissues, hyperplastic AECs in mildly fibrotic tissues and metaplastic bronchiolar epithelial cells in parenchymal scars, and arrows indicate interstitial fibroblasts. **b** OAT expression in lung tissues was measured by immunoblotting. OAT bands were quantified using densitometry. *p < 0.05 versus the control subjects. An anti-OAT antibody was used to immunoblot equal quantities of proteins obtained from lung lysates. The blots were stripped and reprobed with an anti-β-actin antibody. Lung tissues were obtained from patients with IPF and control subjects who underwent thoracic surgery. **c**. OAT was not expressed in inflammatory cells in bronchoalveolar lavage fluid. Magnification, 1000×. **d** OAT expression was induced by BLM. BLM was administered into the tracheas of wild-type mice that were subsequently sacrificed on Day 21. Strong OAT expression was observed in fibrotic areas. Magnification, 100×; inset magnification, 1000×. OAT was not expressed in inflammatory cells. Magnification, 1000×.

Immunohistochemistry was used to evaluate OAT expression in lung tissues from patients with IPF and control subjects. OAT expression was localized to interstitial fibroblasts in lungs affected by IPF, and it was not expressed in hyperplastic alveolar or metaplastic bronchiolar epithelial cells (Fig. 1a and Supplementary Fig. 2). OAT expression was stronger in areas of advanced remodeling than in mildly fibrotic areas (Fig. 1a). Western blotting also showed that OAT levels were significantly increased in lung homogenates from patients with IPF compared to those from control subjects (Fig. 1b). Immunocytochemical staining did not reveal OAT expression in BALF cells (Fig. 1c). BLM is often used to establish in vivo lung fibrosis models^19^. Immunohistochemical staining showed that OAT was strongly expressed in fibrotic areas of mouse lungs (Fig. 1d). Taken together, these results indicate that OAT protein levels are elevated in fibrotic lungs of IPF patients and BLM-treated mice, particularly in the interstitial fibroblasts of severely fibrotic areas.

### Increased OAT expression in IPF fibroblasts

**Fig. 2:**
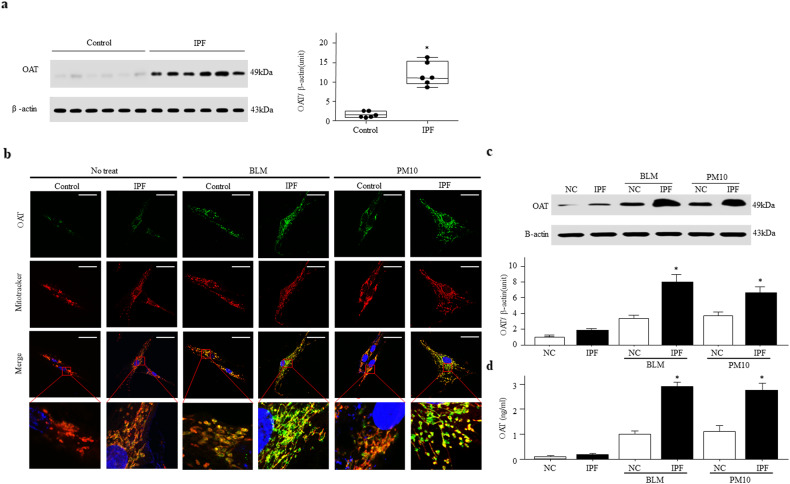
OAT expression is increased in the cytoplasm and mitochondria of fibroblasts under oxidative stress. **a** OAT expression levels in fibroblasts derived from the lung tissues of six IPF patients and six control subjects were measured by immunoblotting. OAT bands were quantified by densitometry. *p < 0.05 versus the control subjects. **b** Confocal analysis of MitoTracker Red and OAT colocalization in fibroblasts from lung tissues of patients with IPF and controls after treatment with 50 µg/mL PM 10 for 24 h. Magnification, 1000×. Scale bar, 50 μm. The insets show higher magnifications (10 × 50 μm rectangles) of the indicated regions of interest. **c** OAT levels in fibroblasts and **d** their culture supernatants after exposure of the cells to oxidative stress-inducing BLM and PM10. The data are presented as the means ± standard errors of the means (SEMs; *n* = 4). *p < 0.05 versus the BLM- or PM10-treated control group.

As OAT was localized to interstitial fibroblasts, we measured OAT expression levels in IPF and control fibroblasts. The OAT expression levels in IPF fibroblasts were significantly higher than those in control fibroblasts (Fig. 2a). Excessive oxidative stress is considered to play a major role in the progression of lung fibrosis^21^. Particulate matter (PM) induces lung inflammation and fibrosis by inducing ROS generation^22,23^. Moreover, exposure to ambient PM is associated with the accelerated decline of lung function in IPF^24^. When fibroblasts were exposed to agents that induce oxidative stress, such as particulate matter (PM)10 or BLM, OAT expression was higher than in fibroblasts that were not exposed to these agents (Fig. 2b, c). As expected, OAT expression was localized to mitochondria in control fibroblasts. Interestingly, some OAT expression was observed in the cytoplasm outside the mitochondria (green, merged image) of IPF fibroblasts that were exposed to PM10 or BLM (Fig. 2b). Consistent with the immunofluorescence data, OAT levels in cell lysates were significantly increased by treatment with PM10 or BLM (Fig. 2c). Surprisingly, OAT levels were increased in the culture supernatants of IPF fibroblasts treated with PM10 or BLM (Fig. 2d). These data suggest that under excessive oxidative stress, IPF fibroblasts increase OAT protein production, and some protein is released into the extracellular space.

### Increased OAT levels in BALF are correlated with a decline in lung function and decreased survival of patients with IPF

**Fig. 3:**
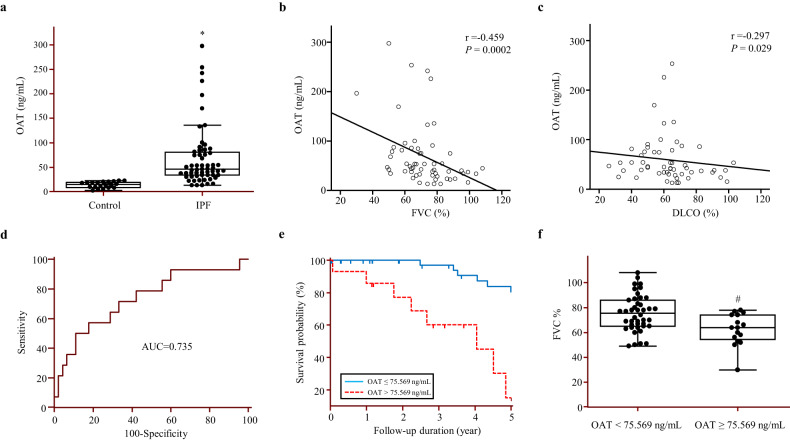
The OAT concentration in bronchoalveolar lavage fluid (BALF) is associated with lung function and the survival of patients with IPF. **a** OAT protein levels in BALF were quantified using enzyme-linked immunosorbent assays (ELISAs). The data are expressed as medians with 25% and 75% quartiles. *p < 0.05 versus the control group. **b** Correlations between BALF OAT levels and forced vital capacity (FVC; % pred.) and **c** between BALF OAT levels and diffusing capacity of the lung for carbon monoxide (% pred.) in patients with IPF. **d** Receiver operating characteristic curve analysis to determine the optimal OAT concentration that could be used as cutoff values for predicting survival in 59 patients with IPF. **e**. Comparison of OAT level survival curves using Kaplan–Meier plots. The survival rate was markedly lower in the group with an OAT level ≥75.659 ng/mL (red line) than in the group with an OAT level <75.659 ng/mL (blue line; hazard ratio, 29.53; 95% confidence interval, 7.29–119.55; *p* < 0.0001). **f** Comparison of FVC in terms of BALF OAT levels. The data are expressed as medians with the 25% and 75% quartiles. #*p* < 0.05 versus the OAT < 75.659 ng/mL group.

Next, we used ELISAs to measure OAT levels in the BALF of control subjects and patients with IPF, and these groups were approximately matched according to age and sex. OAT levels in the BALF of patients with IPF (*n* = 59) were significantly higher than those in the BALF of control subjects (*n* = 20; Fig. 3a). To determine the clinical implications of high OAT levels in patients with IPF, we performed correlation analyses between lung function parameters and BALF OAT levels. Surprisingly, OAT levels in BALF were significantly inversely correlated with FVC and DLCO values (Fig. 3b, c).

During the 5 years of observation, 14 of 59 patients died. The ROC curves demonstrated that an OAT level of 75.659 ng/mL in BALF was optimal for separating survivors from nonsurvivors (area under the curve, 0.735; *p* = 0.0083; Fig. 3d). In terms of predicting mortality, high levels of OAT (75.659 ng/mL) exhibited a sensitivity of 57.14% and specificity of 82.22%. Kaplan–Meier analysis was performed on 59 patients with IPF who were followed up over 5 years to compare the survival of those with low (>5.659 ng/mL) and high (≤75.659 ng/mL) levels of OAT. The survival rate of patients with an OAT level >75.659 ng/mL was significantly lower than that of patients with an OAT level ≤75.659 ng/mL (HR, 29.53; 95% CI, 7.29–119.55; *p* = 0.0008; Fig. 3e). The two groups of IPF patients distinguished using an OAT cutoff of 75.659 ng/mL had significantly different FVC values (*p* = 0.043; Fig. 3f and Supplementary Table). These data suggest that in patients with IPF, OAT levels reflect a decline in lung function and can be used as a prognostic indicator.

### OAT regulates the production of collagen, fibronectin, and α-SMA by fibroblasts

**Fig. 4:**
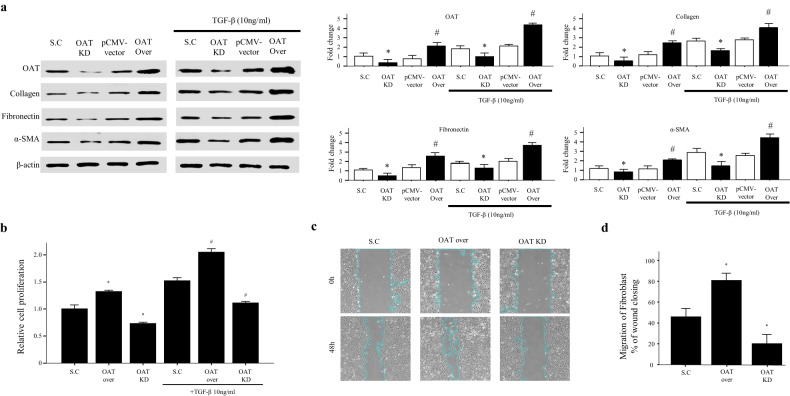
OAT regulates major components of the extracellular matrix and fibroblast proliferation. **a** Effect of knockdown and overexpression of OAT on collagen, fibronectin, and alpha-smooth muscle actin expression in lung fibroblasts. Protein expression was quantified using immunoblotting and densitometry (*n* = 4 per group). **b** Proliferation was assessed using a water-soluble tetrazolium assay. **c** Fibroblast migration was assessed using a wound-healing assay. **d** Quantification of migration. The data are expressed as the means ± SEMs (*n* = 4). *p < 0.05 versus the scramble or BLM-treated scramble group. #*p* < 0.05 versus the pCMV vector group or BLM-treated pCMV vector group.

Because OAT is highly expressed in interstitial fibrotic areas of lungs affected by IPF, we hypothesized that it could regulate the production of ECM components, such as collagen, fibronectin, and α-SMA. Interestingly, the expression of collagen I, fibronectin, and α-SMA was significantly increased in OAT-overexpressing cells treated with or without TGF-β1 (Fig. 4a). Next, we investigated the effect of OAT treatment on fibroblast proliferation. OAT overexpression increased the level of fibroblast proliferation, whereas OAT knockdown decreased fibroblast proliferation, particularly after treatment with TGF-β1 (Fig. 4b). Furthermore, OAT knockdown inhibited migration and increased the time needed for wound healing, while OAT overexpression significantly enhanced migration. (Fig. 4c, d). Taken together, our data demonstrate that OAT regulates both the constitutive and TGF-β1-induced expression of collagen, fibronectin, and α-SMA by fibroblasts as well as the proliferation of fibroblasts.

### OAT knockdown inhibits TGF-β1-induced Smad-dependent and non-Smad signaling pathways

**Fig. 5:**
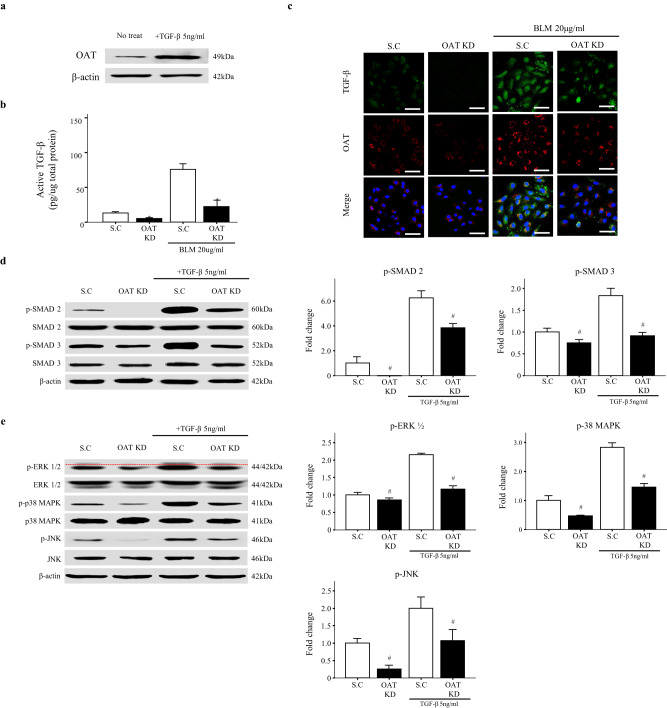
OAT knockdown and overexpression alters transforming growth factor (TGF)-β1 activity in fibroblasts. **a** Effect of TGF-β1 on OAT expression by fibroblasts. **b** Active TGF-β1 levels in fibroblast lysates were quantified using ELISAs. The data are expressed as the means ± SEMs (*n* = 4). *p = 0.05 versus the BLM^+^/OAT scramble group. **c** Expression of active TGF-β1 as determined by immunofluorescence staining. Magnification, 400×. Scale bar, 50 μm. **d** The levels of Smad-dependent signaling pathway proteins (i.e., Smad2 and Smad3) and **e** non-Smad-dependent signaling pathway proteins (i.e., extracellular signal-regulated kinase, c-Jun N-terminal kinases, and mitogen activated protein kinase) were quantified by immunoblotting. Red dotted lines are used to indicate the separation of p-ERK1 and ERK2. Independent experiments were analyzed using densitometry. The data are expressed as the means ± SEMs (*n* = 4). *p < 0.05 versus the scramble or BLM-treated scramble group, and #*p* < 0.05 versus the scramble group or the TGF-β1^+^/scramble group.

TGF-β1 is key for the development of lung fibrosis. First, we found that TGF-β1 increased fibroblast OAT expression (Fig. 5a). We quantified the level of active TGF-β1 in BLM-treated fibroblasts using ELISAs. As expected, the level of active TGF-β1 was increased in BLM-treated control fibroblasts. However, the level of active TGF-β1 was not increased in OAT-knockdown fibroblasts in response to BLM (Fig. 5b). Immunofluorescence staining also showed decreased TGF-β1 expression in BLM-treated OAT-knockdown cells (Fig. 5c). Because OAT overexpression and knockdown regulate active TGF-β1 production, we investigated changes in the levels of downstream molecules in the TGF-β1 signaling pathways (i.e., the Smad2, Smad3, and non-Smad signaling pathways). When OAT-knockdown cells were treated with TGF-β1, we observed a decrease in the phosphorylation of Smad2 and Smad3 compared to controls (Fig. 5d). TGF-β1 also activated non-Smad signaling pathways, including the ERK-, JNK-, and p38 MAPK-mediated signaling pathways. Knockdown of OAT also decreased the TGF-β1-mediated phosphorylation of ERK, JNK, and p38 MAPK (Fig. 5e). These data suggest that OAT can regulate the expression of active TGF-β1 and that inhibition of OAT inhibits TGF-β1-induced Smad-dependent and non-Smad signaling pathways.

### Changes in OAT expression levels regulate proline biosynthesis in vitro

**Fig. 6:**
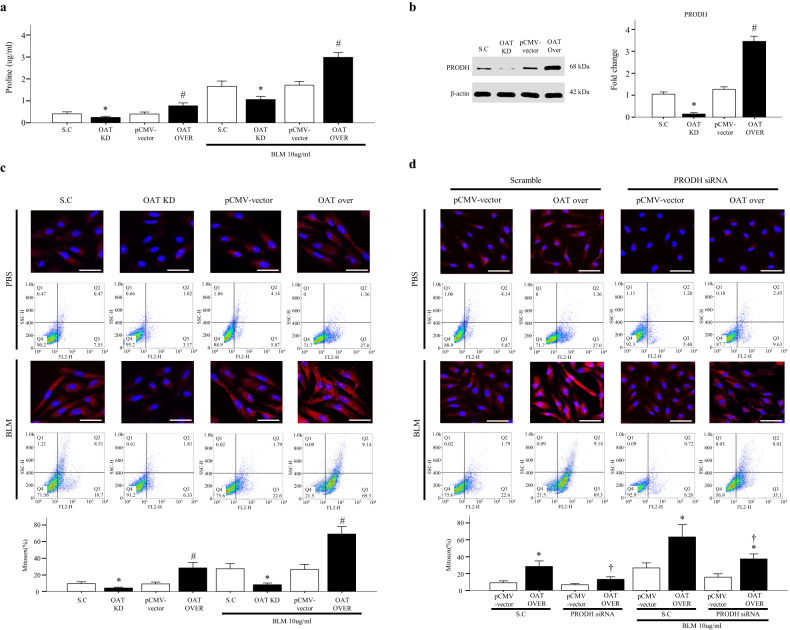
OAT knockdown and overexpression regulate proline dehydrogenase (PRODH)-mediated mitochondrial reactive oxygen species (ROS) production. **a** Proline levels and **b** PRODH expression in OAT-knockdown and OAT-overexpressing lung fibroblasts. **c**, **d** Fluorescence intensities of mitochondria-derived ROS as measured using a MitoSOX Red probe. Magnification, 400×. Scale bar, 50 μm. MitoSOX Red specifically targets mitochondrial ROS, which appear red. The flow cytometry data are quantitative. The data are presented as the means ± standard errors of the means (SEMs; *n* = 4). *p < 0.05 versus the scrambled RNA (scramble) or BLM-treated scramble group. #*p* < 0.05 versus the pCMV vector group or BLM-treated pCMV vector group. ^†^*p* < 0.05 versus the PRODH knockdown/OAT-overexpressing cells or the BLM-treated PRODH knockdown/OAT-overexpressing cells.

As OAT plays a key role in the proline metabolism pathway, we investigated whether changes in OAT expression affected proline production. ELISAs were used to measure proline levels in OAT-overexpressing and OAT-knockdown cells treated with or without BLM. We found that the proline levels were significantly increased in OAT-overexpressing cells compared to control and OAT-knockdown cells (Fig. 6a). Furthermore, in the presence of BLM, proline production was dramatically increased in OAT-overexpressing cells, but OAT knockdown significantly decreased proline production (Fig. 6a). These data confirm that OAT is a critical enzyme for proline biosynthesis. In addition, our data suggest that OAT overexpression significantly enhances proline production, especially in the presence of fibrogenic stimuli.

### OAT increases mitochondrial ROS production by upregulating PRODH levels

OAT plays a key role in converting ornithine into proline in the mitochondrial proline biosynthetic pathway^13^. Mitochondrial PRODH is the rate-limiting enzyme of proline catabolism; PRODH converts L-proline to pyrroline-5-carboxlate^25^. First, we investigated whether changes in OAT expression regulated PRODH; we used Western blotting to measure PRODH protein levels in OAT-overexpressing and OAT-knockdown cells. PRODH levels were decreased in OAT-knockdown cells but significantly increased in OAT-overexpressing cells OAT (Fig. 6b). PRODH produces ROS as a byproduct of proline oxidation^26^; therefore, we used MitoSOX Red to measure mitochondrial ROS levels in OAT-overexpressing and OAT-knockdown fibroblasts that were treated with BLM or not. OAT overexpression dramatically increased the level of mitochondrial ROS, whereas OAT knockdown significantly attenuated ROS production (Fig. 6c). Additionally, PRODH knockdown significantly decreased ROS production, even in OAT-overexpressing cells (Fig. 6d). These data demonstrate that changes in OAT expression may modulate mitochondrial ROS generation via PRODH regardless of whether the cells are exposed to fibrogenic stimuli.

### L-canaline suppresses BLM-induced lung inflammation and fibrosis

To inhibit OAT, L-canaline, which is a well-known irreversible inhibitor of OAT that prevents proline production, was used in vitro^27,28^. A previous report showed that L-canaline has a half-maximal inhibitory concentration of 250 nM, but its cytotoxicity has not been evaluated^29^. Therefore, first, we determined the cytotoxicity of L-canaline. Among the concentrations that were tested, 100 nM L-canaline did not show cytotoxicity (Supplementary Fig. 3).

**Fig. 7:**
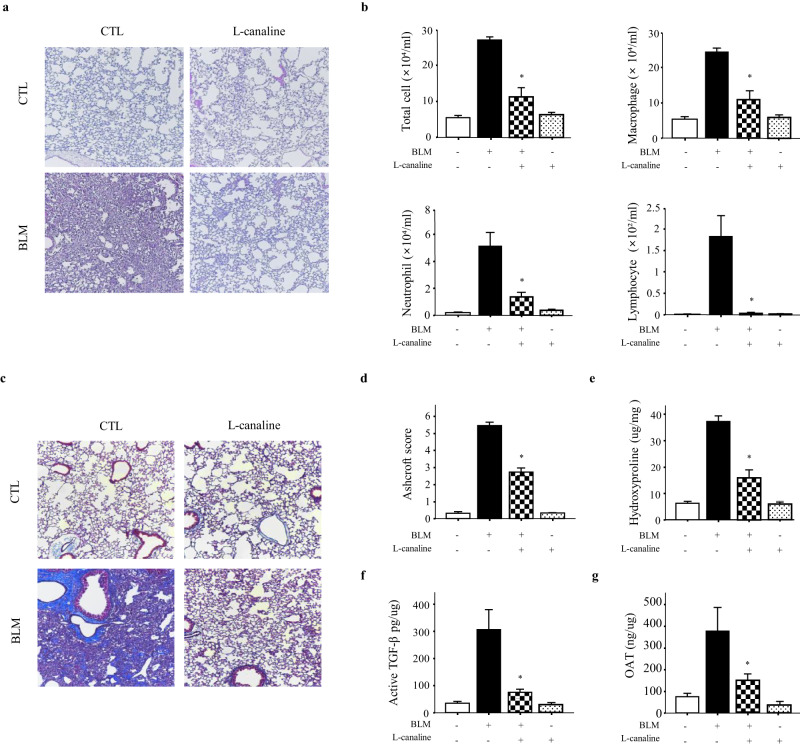
The OAT inhibitor L-canaline attenuates BLM-induced lung inflammation and fibrosis in a mouse model. **a** H&E staining of mouse lungs after 100 nM L-canaline was intranasally administered on Days 8–12. Lung samples were collected on Day 21. Magnification, 100×. **b** Cell counts in BALF collected on Day 21. Differences between BALF cell counts were analyzed based on 500 cells stained with Diff-Quik (*n* = 8 per group). **c** Masson’s trichrome staining. Magnification, 100×. **d** Lung fibrosis as quantified using the Ashcroft score (*n* = 8 per group). **e** Collagen measurements from hydroxyproline assays of control and BLM-treated mouse lungs, with and without L-canaline (*n* = 8 per group). **f** Active TGF-β1 and **g** OAT levels in control and BLM-treated mouse lungs, with and without L-canaline (*n* = 8 per group). Active TGF-β1 and OAT levels in lung lysates were quantified using ELISAs. The data are expressed as the means ± SEMs. *p < 0.05 versus the BLM-treated group.

In our in vitro study, 100 nM L-canaline decreased the protein expression levels of OAT, collagen, fibronectin, and α-SMA in lung fibroblasts (Supplementary Fig. 4). Next, we investigated whether inhibiting OAT could suppress fibrosis in the lungs of BLM-treated mice. The OAT inhibitor L-canaline was intranasally administered 8–12 days after treatment with BLM, and lung samples were evaluated on Day 21. H&E staining showed that L-canaline prevented the BLM-induced disruption of lung structure (Fig. 7a). In addition, analyses of BALF showed that L-canaline significantly attenuated lung inflammation and decreased the numbers of macrophages, neutrophils, and lymphocytes (Fig. 7b). Furthermore, Masson’s trichrome staining and the Ashcroft score showed that L-canaline attenuated collagen deposition-induced lung fibrosis (Fig. 7c, d). Hydroxyproline assays of tissues from BLM-treated mice further confirmed the antifibrotic activity of L-canaline (Fig. 7e). The levels of active TGF-β1 and OAT in the lungs were significantly increased after exposure to BLM but significantly decreased after L-canaline treatment (Fig. 7f, g). These data suggest that the OAT inhibitor L-canaline can protect against BLM-induced lung inflammation and fibrosis.

## Discussion

We showed that OAT expression was increased in lung tissues affected by IPF, particularly in areas of severe fibrotic remodeling, and that the initial OAT levels in BALF may serve as prognostic indicators for IPF. Overexpression and knockdown of OAT in lung fibroblasts demonstrated that OAT could regulate the expression of collagen, fibronectin, and α-SMA in lungs affected by IPF. Moreover, OAT inhibition decreased active TGF-β1 production and inhibited TGF-β1-induced Smad and non-Smad signaling. OAT overexpression increased mitochondrial ROS production via PRODH activation. The OAT inhibitor L-canaline attenuated lung fibrosis in a BLM-treated mouse model. Collectively, these findings suggest that increased OAT levels in lungs affected by IPF contribute to the progression of fibrosis by enhancing TGF-β1 activity and increasing mitochondrial ROS generation; furthermore, inhibition of OAT may alleviate fibrosis.

OAT was principally expressed by interstitial fibroblasts in lung affected by IPF, and it was not expressed by AECs or the metaplastic bronchiolar epithelium (Fig. 1a). Interestingly, the expression levels of OAT were significantly higher in BALF and cultured primary lung fibroblasts from lungs affected by primary than in control samples (Figs. 2a and 3b). Since OAT was not expressed in BALF cells (Fig. 3a), the increased levels of OAT in BALF may be derived from fibroblasts in interstitial fibrotic areas in lungs affected by IPF. In addition, when fibroblasts were exposed to oxidative stress-inducing agents, such as BLM or PM10, OAT expression increased compared to that in unexposed fibroblasts (Fig. 2b, c). Interestingly, some OAT was observed in the cytoplasm outside the mitochondria of IPF fibroblasts that were exposed to BLM or PM10 but not in normal lung fibroblasts (Fig. 2b). Moreover, we detected the OAT protein in the supernatants of cultured primary IPF fibroblasts that were treated with BLM or PM10 (Fig. 2d). It was recently reported that IPF fibroblasts exhibit a senescent phenotype and that mitochondria in senescent cells show increased mitochondrial leakage and decreased mitochondrial membrane potential^30,31^. Thus, we speculate that excessive oxidative stress further damages the mitochondria of senescent IPF fibroblasts. Then, OAT that is overproduced in the mitochondria leaks into the cytoplasm and finally out of the cells. Taken together, our data indicate that upon exposure to factors that induce oxidative stress, the level of the mitochondrial enzyme OAT in IPF fibroblasts increases, and some OAT leaks into the cytoplasm and extracellular space. IPF fibroblasts and myofibroblasts are responsible for the increased deposition of collagen and other extracellular matrix components that trigger lung remodeling and decreased lung function^32^. Therefore, our data suggest that increased OAT levels in IPF fibroblasts may be associated with decreased lung function and poor survival of IPF patients.

We previously reported that OAT mRNA levels were significantly increased in IPF patients from other IPF cohorts^15,16^. Next, we measured the OAT levels in BALF from patients IPF and control subjects, and we observed significantly increased OAT levels in the IPF group (Fig. 3a). Similar to microarray data from other IPF cohorts^16^, our data also suggest that OAT protein levels in BALF are inversely correlated with FVC and DLCO values (Fig. 3b, c). Because FVC and DLCO are reliable parameters for assessing disease severity and progression of ILD^33^, our data suggest that OAT levels in BALF may be as a biomarker of IPF progression. We investigated whether OAT levels could be used as a prognostic biomarker for IPF. ROC curves suggested that an initial BALF OAT level of 76 U/mL could be used as an optimal cutoff point for separating survivors and nonsurvivors over a 5-year follow-up period (Fig. 3d). FVC values were previously proven to be important for predicting mortality among patients with IPF, and low FVC values are associated with disease progression^34^. We found a significant inverse correlation between BALF OAT levels and FVC values, suggesting that these factors can be used as prognostic biomarkers for IPF. However, further studies with larger patient cohorts are needed to confirm this finding.

OAT catalyzes the conversion of L-ornithine into L-proline, which is the principal precursor of collagen^14^. OAT is primarily expressed in the mitochondria of liver and kidney cells^35,36^ and plays a crucial role in proline biosynthesis. We measured proline levels in OAT-overexpressing or OAT-knockdown cells in vitro. Proline levels were significantly increased and decreased, respectively, in these cells (Fig. 6a). This is important, as the findings reflect OAT-specific activity and not an unknown/off-target effect. In addition, our data suggest that changes in OAT expression may regulate the proline metabolism pathway in lung fibroblasts. The overproduction of ECM components is indicative of fibrosis in lungs affected by IPF^37^. Among ECM components, collagen is the most abundant in the lungs of those with IPF. Proline and hydroxyproline are necessary for the processing of modified collagen in the rough endoplasmic reticulum and Golgi apparatus^38^. Therefore, endogenous proline is necessary for collagen synthesis^39^. Arginine, glutamate, and glutamine are potential substrates for proline synthesis^40^, and arginase and OAT are two enzymes that are critically needed^41,42^. Therefore, we hypothesized that the knockdown or overexpression of OAT may alter collagen production. Unexpectedly, we found that OAT knockdown suppressed not only collagen I expression but also fibronectin and α-SMA production (Fig. 4a). In contrast, overexpression of OAT elevated the expression of these components (Fig. 4a). These data suggest that the role of OAT in the proline metabolism pathway in fibroblasts is not limited to collagen biosynthesis.

One of the major characteristics of IPF is uncontrolled fibroblast proliferation. Our results suggest that knockdown of OAT suppresses the proliferation of lung fibroblasts and impairs the migration of these cells (Fig. 4b, d). Therefore, OAT may play a critical role in fibrotic processes characterized by dysregulated fibroblast proliferation and ECM formation.

TGF-β1 is a key mediator of fibrogenesis, both inducing fibroblast proliferation and promoting ECM preservation^43^. Therefore, we investigated the effect of TGF-β1 on OAT-overexpressing and OAT-knockdown fibroblasts. TGF-β1 significantly increased OAT production (Fig. 5a). Additionally, OAT knockdown dramatically decreased, whereas OAT overexpression increased, active TGF-β1 production in the presence of BLM (Fig. 5b). In addition, OAT knockdown inhibited signaling pathways downstream of TGF-β1, including Smad and non-Smad signaling pathways (Fig. 5d, e). Taken together, our results indicate that OAT expression is upregulated by TGF-β1 and that changes in OAT levels regulate active TGF-β1 production by fibroblasts. These data suggest that OAT may interact with TGF-β1 as part of a positive feedback loop.

**Fig. 8:**
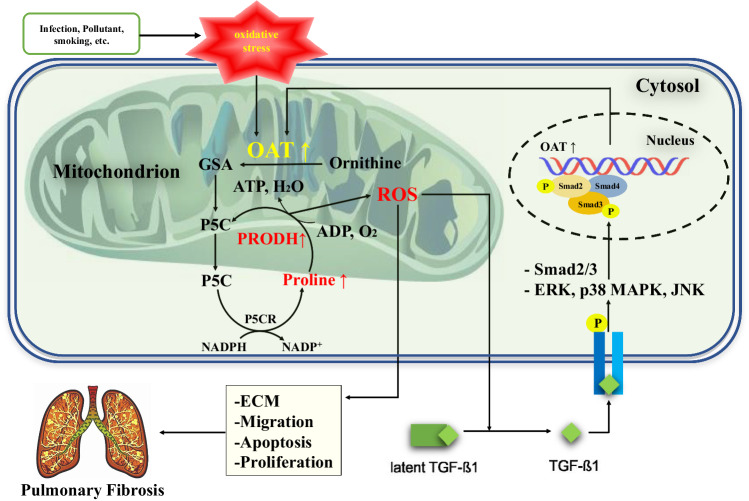
Hypothetical model showing the role of OAT in IPF pathogenesis. Increased expression of OAT activates the proline metabolism pathway and increases the level of PRODH, which generates reactive oxygen species (ROS). The increased ROS levels activate TGF-β1, stimulate fibrotic responses, such as fibroblast proliferation, and modulates ECM production.

Recently, increasing evidence has suggested that mitochondrial dysfunction plays a role in IPF pathology and is associated with increased mitochondrial ROS production^31,44^. Excessive production of ROS plays a role in lung fibrosis by affecting the levels of profibrotic cytokines and growth factors, such as TGF-β1^45^. Mitochondrial ROS are essential for the activation of TGF-β1 signaling^46^, and TGF-β1 may induce ROS generation in senescent cells^47^. It has been suggested that proline metabolism stimulates mitochondrial ROS generation^48,49^. OAT uses ornithine to initiate proline biosynthesis, and proline catabolism is mediated by mitochondrial PRODH^50^, which is a mitochondrial inner membrane-associated enzyme that catalyzes the first (and rate-limiting) step in proline catabolism. During this reaction, electrons are transferred to the mitochondrial electron transport chain for ROS generation^51,52^. We found that PRODH expression was regulated by OAT (Fig. 6b) and that mitochondrial ROS levels were increased by OAT overexpression and diminished by OAT inhibition (Fig. 6c). Moreover, PRODH1 knockdown significantly decreased mitochondrial ROS levels, even in OAT-overexpressing cells (Fig. 6d), indicating that ROS generation by OAT depends on PRODH. As ROS directly activate fibroblasts and regulate interstitial ECM component production^53^ and TGF activity^54^, our data can be explained, at least in part, by the regulatory effect of OAT on ROS generation. Taken together, our results indicate that increased expression of OAT activates the proline metabolism pathway and increases the level of PRODH in lungs affected by IPF, in turn leading to excessive ROS production and establishing a self-propagating, profibrotic signaling loop (Fig. 8).

We investigated whether inhibition of OAT could prevent experimentally induced lung fibrosis. We found that BLM-induced increases in lung inflammation and fibrosis were significantly attenuated by the OAT inhibitor L-canaline (Fig. 7b, d). Collagen production decreased when L-canaline was administered to BLM-treated mice (Fig. 7e). We did not investigate how L-canaline exerted its anti-inflammatory effects. However, OAT inhibition may alter the products of L-arginine metabolic pathways, such as polyamine and nitric oxide, which can stimulate inflammation^55^.

Recently, Liu et al. reported that upregulation of OAT stimulates the proliferation and metastasis of non-small cell lung cancer by upregulating miR-21^56^. In addition, Zigmond et al. found that OAT is overexpressed in hepatocellular carcinoma and that OAT inhibition suppresses the development of hepatocellular carcinoma cells^57^. Combined with our data, these results suggest that OAT may play an important role in the metabolism of rapidly proliferating cells, such as fibroblasts in patients with IPF, as well as in some cancers.

Our study had certain limitations. We did not explore how the OAT protein is secreted into the lungs. Recently, Risha et al. reported that OAT is present in microvesicles and exosomes in cancer cells^58^. As exosomes may be found in BALF^59^, we suspect that OAT may be an exosomal component of BALF in patients with IPF. However, further studies are needed. We did not investigate whether the increased proline levels that are induced by OAT overexpression are used to synthesize collagen. Additionally, the detailed mechanism by which BLM or PM10 upregulates OAT expression remains to be elucidated. Moreover, the multiplicity of substrates/products of OAT catalysis suggests an array of possible mechanisms, such as important roles of glutamate and alpha-keto-glutamate. However, our current research focused on the inhibitory effect of OAT and its role in fibrosis.

In conclusion, OAT is an important mediator of mitochondrial ROS generation in lungs affected by IPF, and its inhibition may be a useful novel therapeutic strategy for regulating ECM component production and TGF-β1 activity in IPF.

### Supplementary information

Supplementary information
