# Supplementary material for: Role of lung ornithine aminotransferase in idiopathic pulmonary fibrosis: regulation of mitochondrial ROS generation and TGF-β1 activity
Source: Exp Mol Med. 2024 Feb 28;56(2):478–90. doi: 10.1038/s12276-024-01170-w (PMC10907606; doi:10.1038/s12276-024-01170-w)
Supplement: Supplementary file 1 — Supplementary information [file 12276_2024_1170_MOESM1_ESM.pdf]

Role of lung ornithine aminotransferase in Idiopathic Pulmonary Fibrosis: regulation of mitochondrial ROS generation and TGF-β1 activity

Authors: Jong-Uk Lee[1], Ki Sung Song[1], Jisu Hong[1], Hyesun Shin[1], Eunji Park[1], Junyeong Baek[1], Shinhee Park[1], Ae-Rin Baek[1], Junehyuk Lee[1], An Soo Jang[1], Do Jin Kim[1], Su Sie Chin[2], U-Jin Kim[3], Sung Hwan Jeong[4], Sung Woo Park[1]*

[1]Division of Allergy and Respiratory Medicine, Department of Internal Medicine, Soonchunhyang University Bucheon Hospital, 14584, Gyeonggi-Do, South Korea.

[2]Department of Pathology, Soonchunhyang University Bucheon Hospital, 14584, Gyeonggi-Do, South Korea.

[3]Department of Internal Medicine Environmental Health Center Kangwon National University, Gangwondaehakgil, Chuncheon-si, Gangwon-do, South Korea.

[4]Department of Allergy, Pulmonary and Critical Care Medicine, Gachon University, Gil Medical Center, Incheon, South Korea.

Supplementary information

Supplementary Table. Clinical characteristics of the patients with IPF classified according to the levels of OAT

	OAT ≤ 75.569 ng/mL (lower)	OAT > 75.569 ng/mL (higher)
No.	43	16
Age (year)	63 (59.22-68.81)	64.77 (56.43-68.65)
Male sex	31 (72.1)	9 (56.25)
Ever Smoker	20 (23.3)	2 (12.5)
BMI, kg/m2	23.68 (22.19-25.97)	25.11 (22.46-26.13)
Follow up duration (year)	5 (2.51-5)	2.45 (1.11-3.77)
Deaths	6 (14.0)	8 (50.0)
FVC (% pred)	75.5 (65.25-85.25)	64 (55.25-74) *
DLCO (% pred)	64 (51-73.75)	60 (53-66.5)
Treatment status		
Anti-fibrotic agents	16 (37.2)	4 (25.0)
Anti-inflammatory agents	25 (58.1)	10 (62.5)
None	10 (23.3)	3 (18.8)
OAT(ng/mL)	38.46 (29.37-49.53)	116.79 (87.13-204.11) *

Data are presented as median median (25th-75th percentile) or number (%).

* : P < 0.05 compared with OAT ≤ 75.569 ng/mL

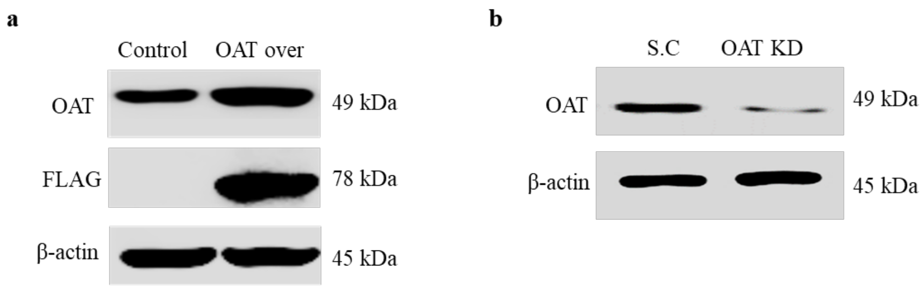

Supplementary Fig 1. Generation of OAT overexpressing and knockdown stable cells.

a. Overexpression of OAT-FLAG and **b.** OAT knockdown stable cells were confirmed by immunoblotting analysis.

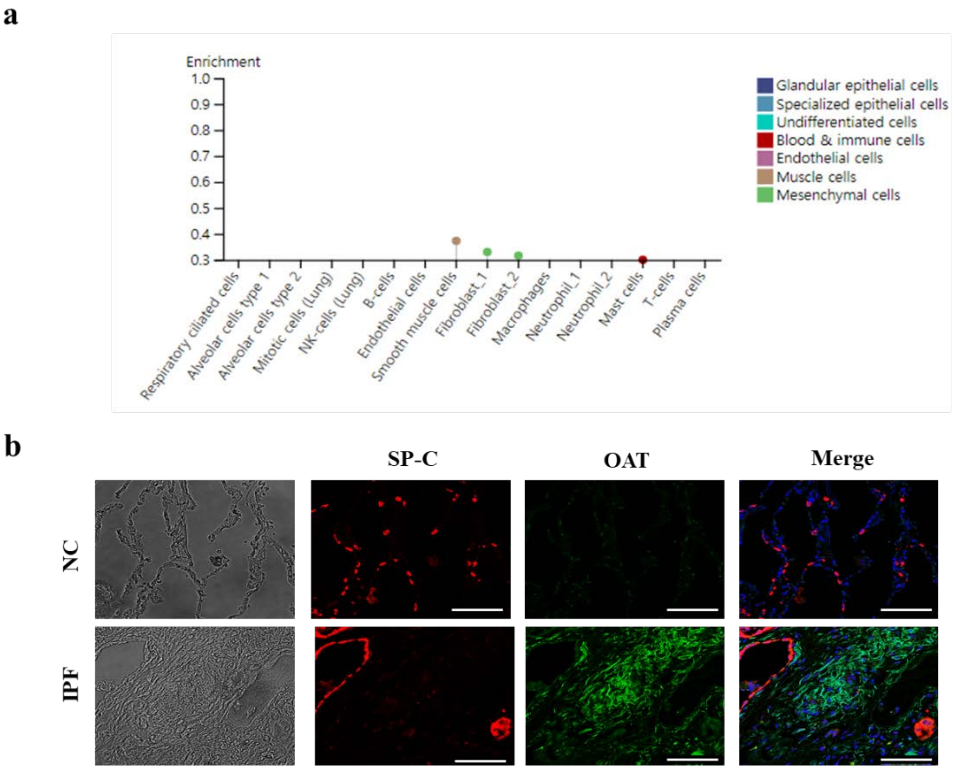

Supplementary Fig 2. Human protein atlas database analysis and double immunofluorescence staining analysis to identify OAT expression.

(a) OAT expression atlas of human lung in normal control. (b) Representative double immunofluorescence-stained images of IPF and control lung tissues. OAT and Surfactant protein C (SP-C) were stained using PE- (red) and FITC-conjugated antibodies (green), respectively. A proportion of alveolar type II cells showed staining for both OAT and SP-C (magnification, 400×)

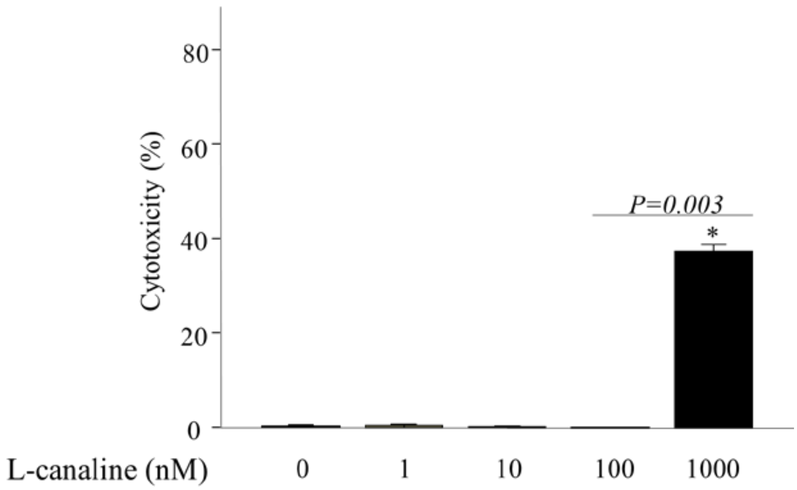

Supplemental Fig 3. Evaluation of mouse alveolar epithelial cell (C57MG) **cytotoxicity at different concentration of L-canaline by the Lactate dehydrogenase (LDH) assay.**

C57MG cells were treated with L-canaline (0-1000 nM) for 24 h in DMEM medium with 0.5% FBS. LDH assay (Cat.88954, Thermo Fisher Scientific, Waltham, MA) followed the manufacturers' instructions. Data are presented as the mean cytotoxicity value ± standard deviation from one representative experiment of four independent experiments. *P<0.05.

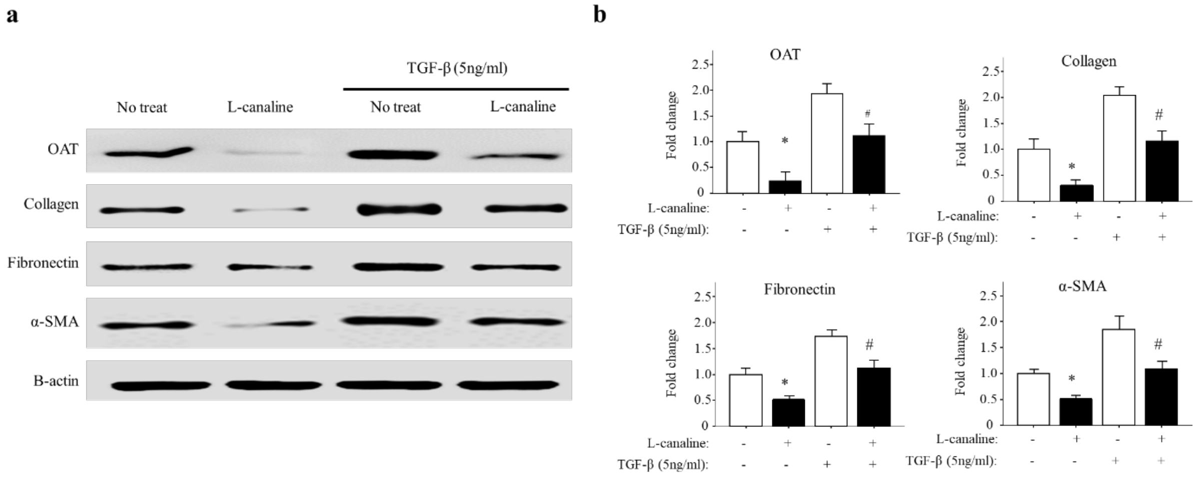

Supplementary Fig 4. The OAT inhibitor L-canaline suppresses the expression of major components of the extracellular matrix in fibroblasts

Effect of the OAT inhibitor L-canaline on OAT, collagen, fibronectin, alpha-smooth mus cle actin, and MyoD expression in lung fibroblasts. **a.** Protein expression was quantified usingimmunoblotting and **b.** densitometry (n = 4 per group). *p < 0.05 versus the scram ble group, and #p < 0.05 versus the L-canaline^{+}/scramble group.
